# Study protocol of a randomized trial of STRIPES: a schoolyear, peer-delivered high school intervention for students with ADHD

**DOI:** 10.1186/s40359-023-01291-3

**Published:** 2023-09-05

**Authors:** Fiona L. Macphee, Stephanie K. Brewer, Margaret H. Sibley, Paulo Graziano, Joseph S. Raiker, Stefany J. Coxe, Pablo Martin, Shauntal J. Van Dreel, Mercedes Ortiz Rodriguez, Aaron R. Lyon, Timothy F. Page

**Affiliations:** 1 Center for Child Health, Behavior, and Development, Seattle Children’s Research Institute, Seattle, WA USA; 2 Department of Psychiatry & Behavioral Sciences, School of Medicine, University of Washington, 6200 NE 74th St, Suite 100, Seattle, WA 98115 USA; 3 Department of Psychology, Florida International University, Miami, FL USA; 4 Department of Management, H. Wayne Huizenga College of Business and Entrepreneurship Nova Southeastern University, Florida, USA

**Keywords:** Psychotherapy, ADHD, Attention-Deficit/Hyperactivity disorder

## Abstract

**Background:**

Youth with ADHD are at risk of academic impairments, dropping out of high school, and dysfunction in young adulthood. Interventions delivered early in high school could prevent these harmful outcomes, yet few high school students with ADHD receive treatment due to limited access to intervention providers. This study will test a peer-delivered intervention (STRIPES) for general education 9th grade students with impairing ADHD symptoms.

**Methods:**

A type 1 hybrid effectiveness-implementation design will be used to evaluate the effectiveness of STRIPES and explore the intervention’s implementability. Analyses will test the impact of STRIPES vs. enhanced school services control on target mechanisms and determine whether differences in basic cognitive profiles moderate intervention response. The acceptability and feasibility of STRIPES and treatment moderators will also be examined.

**Discussion:**

This study will generate knowledge about the effectiveness and implementability of STRIPES, which will inform dissemination efforts in the future. A peer-delivered high school intervention for organization, time management, and planning skills can provide accessible and feasible treatment targeting declines in academic motivation, grades, and attendance during the ninth-grade year.

**Trial registration:**

This study is registered on OSF Registries (10.17605/OSF.IO/Q8V6S).

## Background

Youth with ADHD experience critical academic impairment during high school (HS; grades 9–12, approximately 14–19 years old), including completing fewer assignments and earning lower grades and test scores [1–5] compared to peers without ADHD. Up to one third of youth with ADHD drop out of HS [6]. These academic problems predict severe dysfunction in young adulthood [7–9]. Students who do not finish HS risk further escalating problems (i.e., criminal behavior, unemployment, addiction, public assistance) [10–12], and young adults with ADHD are at elevated risk for each of these problems [13, 14].

Despite these difficulties, most HS students with ADHD receive no treatment (medication or psychosocial) and are placed in general education [2, 15, 16] despite academic difficulties. Though there are many evidence-based interventions for children with ADHD during elementary school years [17], almost no such interventions occur in HS settings due to structural and resource barriers. For example, elementary teachers typically work in one classroom and oversee behavioral interventions for students with ADHD [18]; however, HS teachers teach over 100 students and have little time for individual student support [19]. Additionally, HS counselor duties include graduation planning, parent liaising, and student scheduling rather than intervention implementation and support [20]. Ancillary intervention staff are needed to provide services [21]; however, funding for staff and services—particularly for students without special education entitlement—are declining [22].

In well-resourced HS settings, providers can offer services using elementary or middle school models [23]. The most promising may be interventions developed for middle schoolers [24] and adapted for HS youth in clinical [25], school [26], and intensive settings [27]. These interventions target two core ADHD deficits: executive functioning (EF) and academic motivation (AM) [28, 29]. They teach organization, time management, and planning (OTP) strategies and include motivational components such as goal setting, contingency management, and strength-based feedback [30]. Despite these approaches’ promise in clinical settings, intervention delivery by HS staff is often impeded [31, 32].

This challenge was demonstrated in a study that showed low success rate for an intervention that involved behavioral consultants identifying a school-staff interventionist for 218 adolescents with ADHD at 114 different schools [32]. Monthly contact between consultants and school-staff interventionists was challenging (38.5% success rate), and only 40.0% of interventionists completed in-person meetings with consultants. Lower success rates were associated with HS (vs. middle school) and general (vs. special) education settings. When surveyed about barriers, school staff highlighted resource problems. Thus, an ongoing challenge is identifying qualified and available interventionists to deliver interventions to general education HS students with ADHD.

### Task shifting to peers as interventionalists

One group of interventionists who are available, qualified, and willing may be older HS student peers. Peers are numerous, free interventionists who possess more time than school staff to devote to service delivery. Additionally, students have ample opportunities to interact with peers during school, and may be highly motivated to deliver interventions to enhance college applications, earn community service hours, and serve as a learning experience. This lower-resource model may be fitting for general education students with ADHD who do not require intensive intervention.

HS students can deliver a range of interventions to peers with fidelity [33–36]. Meta analysis [37] suggests that peer- and adult-delivered disruptive behavior interventions targeting aggression produce similar effect sizes to professional delivered models. Peers play a central role in the lives of HS students [38] and can serve as salient reinforcers in behavioral therapy [39]. A peer-delivered intervention may provide social and mental health benefits to students with ADHD who are at risk for peer rejection and often have few friends [40].

### Ninth grade as a strategic window for intervention

Ninth grade is an important time for students with ADHD to access services. Performance during this year is a robust predictor of HS dropout [41]. Further, declines in grade point average (GPA) [42], self-esteem [43], and adjustment to HS are prevalent among adolescents at this age, and this is prominent in students with ADHD, whose 9th grade year is the trough of their academic performance [4]. Interventions for ADHD may be particularly effective when delivered in 9th grade. In previous work [27], our team delivered a summer program to rising 6th and 9th graders with ADHD. The intervention had greater effects on symptoms and functioning in 9th (vs. 6th) graders. We hypothesize that greater cognitive maturity in 9th graders promoted skill uptake and generalization.

### Development of a peer-delivered intervention

Students Taking Responsibility and Initiative through Peer Enhanced Support (STRIPES) is a peer-delivered intervention for general education 9th grade students with impairing ADHD symptoms that was developed over four years [44]. A stakeholder grounded approach was used to develop STRIPES from existing evidence-based treatments for ADHD [23]. Study 1 (*N* = 18) established initial acceptability (i.e., credibility, bond, satisfaction, perceived helpfulness) and feasibility of the service delivery model (i.e., attendance, fidelity). Qualitative methods were used for consumer perspectives on strengths, weaknesses, and strategies to improve STRIPES. Results indicated that an elective-pullout model best fit student’s needs. Study 2 (*N* = 72) was a randomized controlled trial that compared STRIPES to an active control in three HSs. Each school chose a tailored implementation model (i.e., STRIPES was delivered during lunch at one school, as elective pull-out at another, and after school at another). Peer-retrieval and elective pull-out model features possessed strong consumer fit, and when delivered with a population-fitting implementation model, the low-burden STRIPES intervention met acceptability and feasibility metrics and impacted AM, EF and student outcomes.

### Current study

This project employs a type 1 hybrid effectiveness-implementation design [45], to evaluate the effectiveness of STRIPES while understanding the intervention’s implementability. Efforts to work collaboratively with schools to develop and test school-specific implementation plans will be supported by the Consolidated Framework for Implementation Research (CFIR) [46]. The CFIR identifies five major domains that interact to influence implementation and effectiveness: intervention, outer setting, inner setting, characteristics of individuals, and process. The CFIR will be used to guide STRIPES evaluation and implementation planning by supporting selection of implementation constructs to measure, methods of measurement, and design of implementation strategies.

### Target mechanisms of intervention

**Fig. 1:**
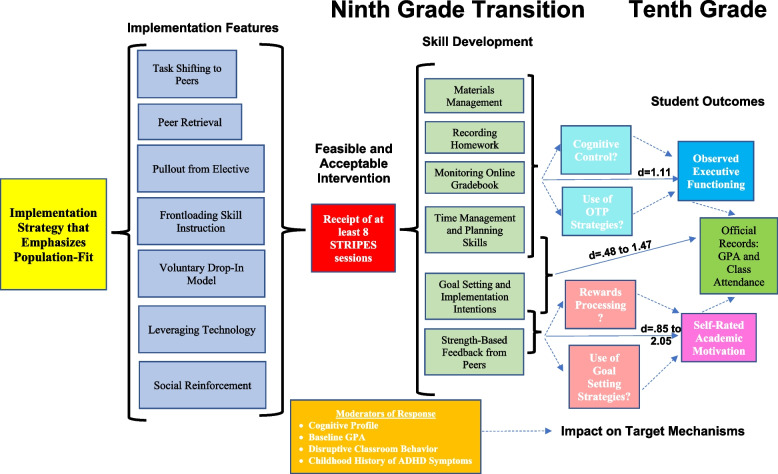
STRIPES logic model Dotted lines are hypothesized effects to be tested. Solid lines are previously established effects to be replicated

We will target two inter-related mechanisms (see Fig. 1): (1) AM and (2) EF, which are linked to academic success in students with ADHD [47, 48]. Hallmarks of ADHD-related HS impairment are related to both EF and AM as reflected by poor work completion and inadequate test preparation [4].

#### Academic motivation

AM mediates the relationship between ADHD-related rewards deficits and grades [49]. With respect to intrinsic motivation, students with ADHD report low academic interest and perceive schoolwork as highly aversive [50, 51]. Deficits in intrinsic motivation are tied to abnormal anticipatory dopamine response [52] — a core deficit in ADHD [53]. Thus, typical academic work feels less intrinsically rewarding to students with ADHD as they experience lower sense of novelty, curiosity, enjoyment associated with it. In HS, these deficits may be prominent due to the repetitive and complex nature of academic tasks [43]. Intrinsic motivation deficits are further hampered by ADHD-related learning problems that increase the aversiveness of schoolwork [54] and are compounded by ADHD-related delay aversion [28] — mental discomfort when tasks contain delayed rewards.

With respect to extrinsic, value-driven motivation, students with ADHD report valuing achievement and mastery less than peers [55–59]. For students with ADHD, a preference for immediate rewards [60] may prevent high valuation of long-term and symbolic reinforcers (i.e., grades). Students with ADHD show reduced sensitivity to future negative consequences [29], which reduces motivation to avoid negative outcomes (e.g., course failure). They also may exert lower academic effort because they perceive a reduced probability of earning high grades, due to years of school failure [61].

#### Executive functioning

Even with adequate AM, persistent difficulties with OTP likely reflect deficits in various domains of EF [62, 63] preventing ultimate academic success. EFs are top-down cognitive processes that help students implement actions in support of goals and suppress counterproductive motivational states [64]. EFs include working memory, response inhibition, and cognitive flexibility. Together, these EFs underlie OTP and goal-directed behaviors like planning, task-initiation, inhibiting unproductive behaviors, and task disengagement. EFs are notably impaired in students with ADHD [28, 65], who show consequent difficulties with OTP [66] and goal pursuit [67, 68].

### Study aims

This study first aims to engage school stakeholders to establish school-specific variations of the STRIPES implementation strategy that attend to developmental, disorder, and context-specific considerations and to document interventionist implementation fidelity (Aim 1). The CFIR will be used to guide the selection of questions for stakeholder interviews about potential barriers and facilitators to the implementation of STRIPES in the school context [46]. A randomized trial will then evaluate the impact of STRIPES (vs. enhanced school services control; SSU +) on target mechanisms including EF, AM, and student outcomes (GPA and class attendance; Aim 2). We will also estimate the extent STRIPES remediates cognitive control and rewards processing deficits, facilitates the development of compensatory skills that overcome the impact of deficits, or both. Aim 3 will explore whether differences in basic cognitive profiles moderate intervention response. The acceptability and feasibility of STRIPES will be examined with consideration of student, peer interventionist, and school stakeholder perspectives, and cost to schools (Aim 4). Lastly, we will investigate ecologically valid treatment moderators (baseline GPA, history of ADHD, disruptive classroom behavior, peer characteristics; Aim 5).

## Methods

### Study timeline

Year 1 will focus on planning and collaborative work with two schools, in Seattle, WA and Miami, FL, to pilot school-specific implementation plans. In years 2 through 4, we will conduct a randomized controlled trial comparing (1) STRIPES to (2) SSU + . In each of the three annual cohorts, 24 9th grade students will be recruited at each of the two schools (12 per condition; total *N* = 144) and will be randomized within school and cohort using permuted block randomization. Teachers who nominate students will obtain written parental permission to nominate, a demographic form, and a DSM-5 ADHD checklist [32]. A core academic teacher completes the same ADHD checklist and impairment measures (IRS; [69, 70]). Ninth grade students will be eligible to participate if they display at least 4 symptoms of inattention or hyperactivity/ impulsivity and elevated academic impairment, defined as meeting two of the following: (1) D or F in an academic class, (2) at least 20% of assignments missing in a class, (3) “3” or higher on the teacher IRS academic impairment item (0-6 scale; [70]), (4) elevated score on the teacher Adolescent Academic Problems Checklist (AAPC; 4 items endorsed “pretty much/very much;” [69]). Students are excluded if placed in special education classes, as this study aims to test a low-cost intervention in general education settings. Students with comorbid learning disabilities will be eligible if placed in general education classes. Peer interventionists will be nominated by teachers and must have at least a 3.0 GPA and no suspensions during the past 12 months. Written parental consent and youth assent will be required [70].

### Study conditions

#### STRIPES

STRIPES aims to deliver eight 30-min sessions per student over 16 weeks [44] via peer interventionists who are trained in aiding in goal setting and affirming positive steps. See Table 1 for a summary of intervention characteristics. Peer interventionists will receive four hours of initial training plus weekly supervision by a school staff sponsor. The school staff sponsor will receive two hours of initial training, weekly consultation with a school mental health liaison, and feedback on fidelity from research staff.

**Table 1:** STRIPES intervention characteristics

Core Intervention Components	Implementation Features
Materials management (EF strategy)	Task shifting to peers to overcome shortage of school staff interventionists
Recording homework assignments (EF strategy)	Peer retrieval to overcome forgetfulness
Consistently monitoring online gradebook (EF strategy)	Pullout from elective to avoid loss of highly valued social time and academic instruction
Time management and planning skills (EF Strategy)	Frontloading skill introduction to increase skill exposure for intermittent attenders
Goal setting and implementation intentions (Motivation strategy)	Voluntary attendance and drop-in model (16 sessions offered with the goal of providing 8)
Strength-based feedback from peers (Motivation strategy)	Leveraging technology (monitoring online gradebook and school academic dashboard)
	Social rather than tangible reinforcement to overcome school resource shortages

#### Comparison

Students assigned to SSU + will be referred to their school counselor with the baseline (BL) assessment of symptoms and presenting problems, and their services received will be tracked.

### Assessment procedures

Ecological school outcomes will be collected at five longitudinal time points (once per quarter in 9^th^ grade and once in 10^th^ grade). Target mechanisms will be assessed off medication at the five longitudinal time points and at BL, post-treatment at the end of 9th grade (FU1), and at the beginning of 10th grade (FU2). Compensatory strategies and cognitive processing measures will be collected at BL, FU1, and FU2.

### Measures

#### Ecological school outcomes

GPA will be calculated by converting academic grades to a 5-point scale (i.e., 4.0 = A to 0.0 = F). Number of class absences will be calculated for each quarter.

#### Compensatory strategies

The academic skills subscale of the Adolescent Academic Problems Checklist [69] is a validated 24-item teacher-report measure of the application of OTP strategies in the classroom [69]. Factors include academic skills, disruptive behavior, and a total score, with strong internal reliability and concurrent validity [27, 69, 71]. The goal setting and planning section of the Self-Regulated Learning Interview Schedule [72] will be converted to self-report.

#### Target mechanisms

##### Functional indices of executive functions

Percentage of classes with recorded homework will be calculated for the last five school days [73]. Bookbag observations will be obtained by research assistants using the Organization Checklist [74]. Missing assignments will be gathered monthly through the online grade portal.

##### Academic motivation

A self-report change ruler measure will be used that rates various aspects of motivation using an 11-point Likert Scale (0 = not at all to 10 = extremely). The measure possesses established psychometric properties with adolescent populations [75] and is sensitive to change in treatment outcome studies for adolescents with ADHD [44, 76]. It also correlates strongly with longer motivational questionnaires but outperforms these measures at predicting behavioral intentions [77]. A validated 22-item adaptation for HS students [78] of the Basic Psychological Needs Scale will measure three conditions that promote intrinsic motivation [79].

##### Cognitive and rewards processing

Three indices of EF will be measured by cognitive tasks: working memory, response inhibition, and cognitive flexibility. Working memory performance will be assessed using the List Sorting Working Memory Test from the NIH Toolbox [80], which shows excellent test–retest reliability and convergent and discriminant validity with other working memory measures [81].

Response inhibition will be measured using a go/no-go task that uses both positively and negatively valenced emotional stimuli [82], shows good convergent validity [83], and is validated for adolescents [84]. Response inhibition will be evaluated using the Flanker Inhibitory Control and Attention Test from the National Institute of Health (NIH) Toolbox. Cognitive flexibility will be measured using the NIH Toolbox Dimensional Change Card Sort Test [80], which shows excellent developmental sensitivity and convergent validity [85]. Risky decision making will be assessed using a computerized Iowa gambling task (Hungry Donkey Task) [86]. This task shows good convergent validity in adolescents [87]. Delayed discounting will be measured using a computerized Choice-Delay Task [60], which shows developmental sensitivity [60] and correlates with symptoms of ADHD [88].

#### Engagement and fit

##### Acceptability

Treatment credibility will be measured using the Client Credibility Questionnaire [89]. Students will rate how logical they found treatment and how confident they were in the treatment using a 3-point scale (0 = not at all to 2 = very much). Participants will complete the Therapist Bond Scale [90], rating the degree to which they enjoyed working with peers during STRIPES using a 4-point scale (1 = not at all like you to 4 = very much like you). Lastly, participants will rate treatment satisfaction using a questionnaire developed for behavioral treatments [91] and adapted for adolescents with ADHD [27, 73]. Respondents indicate satisfaction using a 7-point Likert Scale (1 = Strongly Disagree, 7 = Strongly Agree).

##### Service utilization

A combination of district records collection and structured interviews with the student and the student services/special education team leaders at each school will track service utilization for both groups. Interviews will focus on school services accessed by students, with structured prompts ensuring coverage of multiple service modalities.

#### Potential covariates

Medication use will be monitored using the Services for Children and Adolescents-Parent Interview [92]. We will also measure the following potential covariates at BL: intelligence quotient, parent education level, race/ethnicity, age, gender, parent marital status, and free/reduced lunch.

### Patient and public involvement

No patient involvement in the design of this study.

### Data analysis plan

Analyses will be performed using Mplus version 7 or higher. Missing data will be handled with full information maximum likelihood estimation.

#### Aim 2

The direct effect of STRIPES on each variable (GPA, class attendance, AM, EF) will be evaluated using mixed models [93]. Each outcome will be analyzed with a separate model with time modeled as person-specific (months since BL). We will explore linear, non-linear (i.e., logarithmic, exponential, quadratic), and piece-wise growth models to determine whether there are unique influences of STRIPES on time frames, reflected by slope over time; the best model will be selected using likelihood ratio tests. Effects of time, group, and their interaction will serve as model predictors, and covariates will be included.

#### Aim 3

Aim 3 tests three sets of mediational effects. The mechanisms by which STRIPES affects improvement in student outcome will be evaluated using a structural equation modeling framework in Mplus [94]. Significance of indirect effects will be assessed by bootstrap confidence intervals [95, 96]. In each model, variables that are repeatedly measured (i.e., outcomes, AM, observed EF, rewards processing, motivational strategy, and cognitive control) will be modeled with a mixed model as described in Aim 2. Aim 3a examines the effect of STRIPES on outcomes via AM and observed EF. Aim 3b examines the effect of STRIPES on AM via rewards processing and/or motivational strategy use. Aim 3c examines the effect of STRIPES on observed EF via cognitive control and/or motivational strategy use. For each model, STRIPES predicts the intercept and slope of the mediator model (a paths) and the intercept and slope of the outcome model (c’ paths), while the intercept and slope of the mediator model predict the intercept and slope of the outcome model (b paths).

Current recommendations advise evaluating mediators individually before combining them into a model with simultaneous mediators [97]. For example, Aim 3a will examine AM as a mediator of the STRIPES to outcome relationship in one model, EF as a mediator of the STRIPES to outcome relationship in a second model, and examine both variables as simultaneous mediators in a final model.

#### Aim 4

Analyses will adhere to Consolidated Health Economic Evaluation Reporting Standard recommendations [98]. Costs will be compiled for each trial arm at the student, school, and district levels. Cost-effectiveness analysis is the preferred evaluation method for interventions involving well-defined, non-monetary outcome measures [99]. This method does not require that the effectiveness measure be translated into dollars, and it allows for comparisons across multiple interventions if they have common outcomes of interest. The primary learner outcomes for cost effectiveness analyses will be class attendance and GPA. In cases where one treatment arm is both more costly and more efficacious, incremental cost-effectiveness ratios (ICERs) will be computed. The ICER reports the additional cost of achieving a one-unit improvement in the outcome measures relative to the next least expensive tier. When there are statistically significant differences in outcomes, cost-effectiveness acceptability curves will be constructed to account for variability in the cost and outcome data. These curves yield the probability of one treatment being preferred over another assuming different threshold levels for the value of a unit of outcome improvement [100]. Additional one-way and probabilistic sensitivity analyses will be conducted as needed.

#### Statistical power

**Fig. 2:**
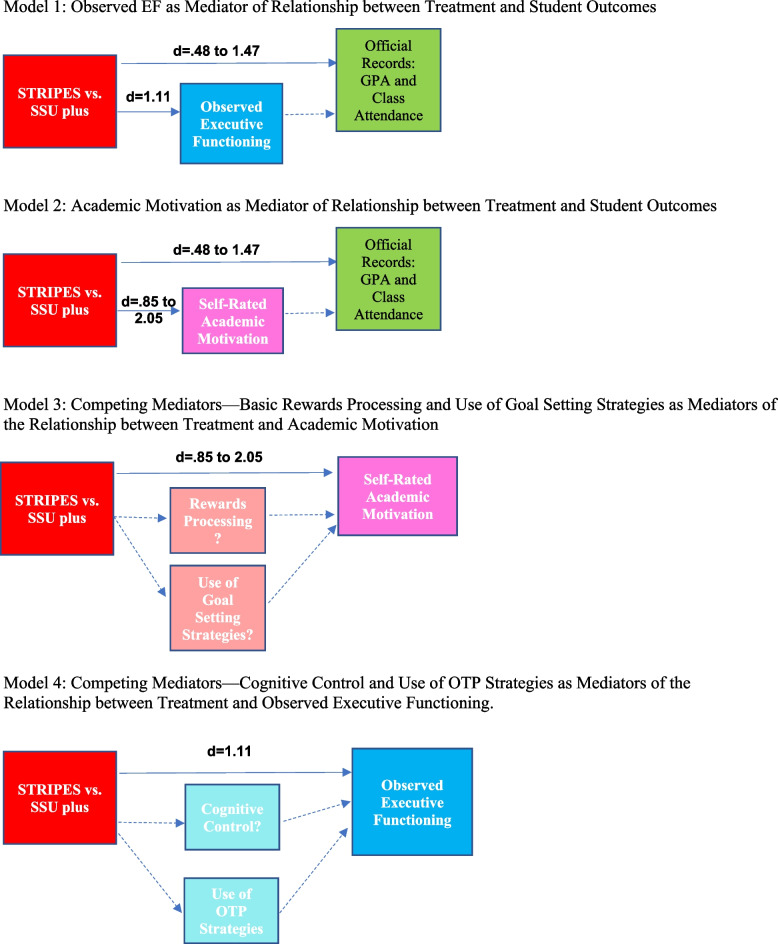
Proposed mediational models in analytic plan

We estimate 5% attrition based on previous studies. Statistical models are outlined in Fig. 2. Aim 2. Based on simulations by Fan [101], we assessed statistical power for mixed models, given a fixed sample size of 144. Based on previous research [44], we expect group differences at later time points to range from medium to very large (*d* = 0.47, 0.85, 1.11, 1.47, 1.69, 2.05 for primary outcomes); Fan [101] found that statistical power exceeded 0.80 with samples of about 100 with medium or larger effects. Aim 3. For the indirect effects proposed in Aims 3a-3c, statistical power estimates are based on sample size calculations by Fritz and MacKinnon [102]. Statistical power for the indirect effect depends entirely on the values of the “a path” (X to M effect) and “b path” (M to Y effect) effect sizes. With a sample size of 144 and using preferred bootstrap methods, we have sufficient power to detect the indirect effect provided both the “a path” and the “b path” are greater than *d* = 0.35. Prior research [44] shows “a paths” for Aims 3a and 3b that range from *d* = 0.85 to 2.05. Aim 4. We will assess statistical power of the cost-effectiveness analysis using methods outlined by Boyd and colleagues [103]. Aim 5. Due to the complexity of estimating statistical power for the mixed models in Aim 5, we base our estimates of power on effects from the previous STRIPES trial [44], which used similar models for analysis with a substantially smaller sample. We used F statistics to estimate the non-centrality parameter, estimated a denominator degrees of freedom based on more observations (144 students each measured 5 times versus 72 students each measured 4 times), and estimated statistical power for a generic F test based on these values. Of the 35 effects examined, the mean power value was 0.61. Seven effects had estimated power > 0.80; based on the non-centrality parameters for these effects, we have adequate power to detect effects as small as Cohen’s *d* = 0.245, which is a small effect size.

## Discussion

Given the increased difficulties experienced by 9th grade students with impairing ADHD symptoms, it is important to identify accessible and feasible interventions that target needed skills. This study will generate knowledge about the effectiveness and implementability of STRIPES, which will inform dissemination efforts in the future.

## Data Availability

Anonymized data will be available on reasonable request (contact corresponding author Stephanie K. Brewer, Ph.D.).

## Abbreviations

ADHD: Attention-Deficit/Hyperactivity Disorder
AM: Academic motivation
BL: Baseline
CFIR: Consolidated Framework for Implementation Research
EF: Executive functioning
FU1: End of 9^th^ grade
FU2: Beginning of 10^th^ grade
GPA: Grade point average
HS: High school
ICER: Incremental cost-effectiveness ratio
NIH: National Institute of Health
OTP: Organization, time management, and planning
SSU +: Enhanced school services control
STRIPES: Students Taking Responsibility and Initiative through Peer Enhanced Support
